# Not Just Another Facial Abscess: Cervicofacial Actinomycosis Uncovered by Skin Biopsy

**DOI:** 10.7759/cureus.91337

**Published:** 2025-08-31

**Authors:** Mindy Kresch-Vatch, Brian A Moreno, Moises Lutwak, Daniel Lutwak, Stanley Skopit

**Affiliations:** 1 Dermatology, Larkin Community Hospital, South Miami, USA; 2 Dermatology, Lake Erie College of Osteopathic Medicine, Bradenton, USA

**Keywords:** actinomyces infection, actinomycosis, chronic cutaneous lesion, clinical dermatology, dermatology consult, dermatology diagnosis, dermatology outpatients, erythema, facial lesion, subcutaneous nodule

## Abstract

Cervicofacial actinomycosis is a rare, chronic bacterial infection that typically arises after mucosal disruption, often due to dental procedures. We present the case of an 18-year-old female patient with a persistent erythematous cheek nodule following wisdom tooth extraction. Initial imaging was non-specific, and prior treatment was deferred due to diagnostic uncertainty. A punch biopsy revealed sulfur granules and histologic features consistent with actinomycosis, though molecular testing detected only commensal flora. Initiation of targeted antibiotic therapy led to clinical improvement. This case underscores the diagnostic value of dermatologic biopsy in atypical post-dental lesions and highlights the limitations of molecular diagnostics in isolating *Actinomyces*.

## Introduction

Actinomycosis is a chronic bacterial infection caused by anaerobic or microaerophilic gram-positive bacilli from the genus *Actinomyces*, organisms that are part of the normal flora of the oral cavity, gastrointestinal tract, and genitourinary tract [1]. Cervicofacial actinomycosis is the most common clinical form of this disease, often presenting as a slowly progressive, firm, and sometimes draining mass of the jaw or face, particularly in the aftermath of dental trauma or oral surgical procedures [2]. Tooth extractions, particularly of lower third molars, are frequently associated with the development of such infections due to mucosal barrier disruption and subsequent bacterial invasion into deeper tissues [3].

Clinically, cervicofacial actinomycosis may mimic a wide range of dermatologic and neoplastic conditions. Patients often present with firm subcutaneous nodules, draining sinuses, or abscesses, and may not exhibit systemic symptoms such as fever or malaise [4]. The classic sulfur granules, yellow colonies of filamentous bacteria, may be observed grossly or microscopically in exudates or biopsy specimens, offering a diagnostic clue [2,5]. However, these granules are not always present, and cultures may often yield only normal flora or false negatives due to the fastidious nature of the organism [5].

Diagnosis is further complicated by the non-specificity of imaging findings and the absence of pathognomonic clinical signs in many cases. Ultrasound, often used as an initial tool in evaluating facial masses, may reveal nonspecific soft tissue abnormalities, and CT or MRI may be needed when deep tissue extension or osteomyelitis is suspected [6]. Importantly, in the dermatologic setting, biopsy remains a critical diagnostic modality, particularly when actinomycosis presents as a cutaneous or subcutaneous lesion without overt dental findings [7].

The management of cervicofacial actinomycosis typically involves prolonged antibiotic therapy. Historically, penicillin for 6-12 months was standard, though more recent studies suggest that shorter courses may be sufficient in cases where adequate surgical drainage or debridement has been performed [8]. First-line therapy includes penicillin or amoxicillin, with alternatives such as doxycycline or clindamycin considered in patients with allergies [9].

Herein, we present the case of an adolescent female patient who developed a fluctuant, erythematous nodule on the lateral jaw following wisdom tooth extraction. The diagnosis of cervicofacial actinomycosis was ultimately established via skin biopsy after initial clinical uncertainty, highlighting the critical role of dermatologic evaluation in atypical post-dental facial lesions.

This case is notable for its delayed diagnosis, the patient’s initial refusal of antibiotics, and the eventual diagnostic confirmation through skin biopsy despite negative molecular findings. It emphasizes how dermatologic evaluation can play a pivotal role in diagnosing deep infections not initially suspected.

## Case presentation

An 18-year-old female patient with no significant past medical history presented to the dermatology clinic for evaluation of a persistent lesion on the right lateral jaw. The lesion first developed approximately four months prior to the presentation, two months after she underwent wisdom tooth extraction. She reported initial induration and significant tenderness, which prompted evaluation by an oral surgeon. At that time, she was prescribed antibiotics, but declined to take them due to uncertainty about the diagnosis. In the interim, she pursued treatment with herbal supplements provided by a Chinese acupuncturist, which she reported reduced the pain but left the lesion more fluctuant. She denied systemic symptoms or drainage.

**Figure 1:**
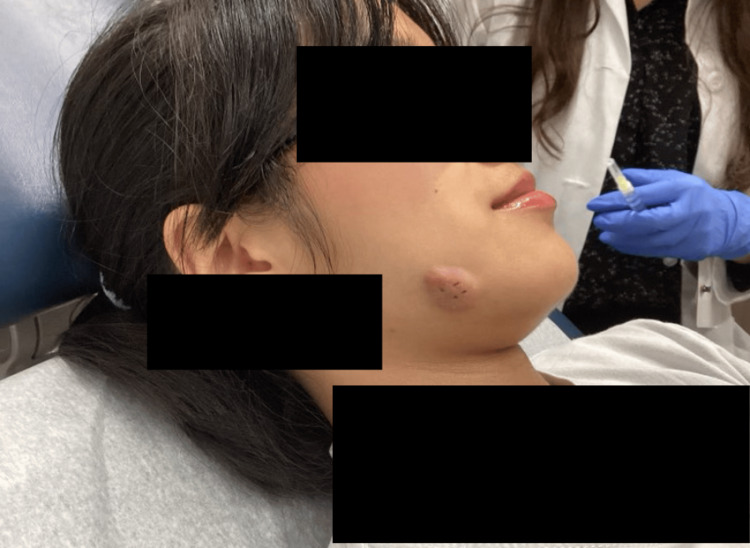
Clinical image of right buccal cheek showing a solitary erythematous fluctuant nodule at initial presentation

Physical examination revealed a solitary erythematous, fluctuant nodule on the right buccal cheek (Figure 1). No mucosal lesions, fistulae, dental abscesses, or intraoral cavities were appreciated on examination. The gingiva appeared intact with no exposed bone or signs of active dental infection. An ultrasound was performed and was notable for nonspecific findings, without evidence of a solid mass.

**Figure 2:**
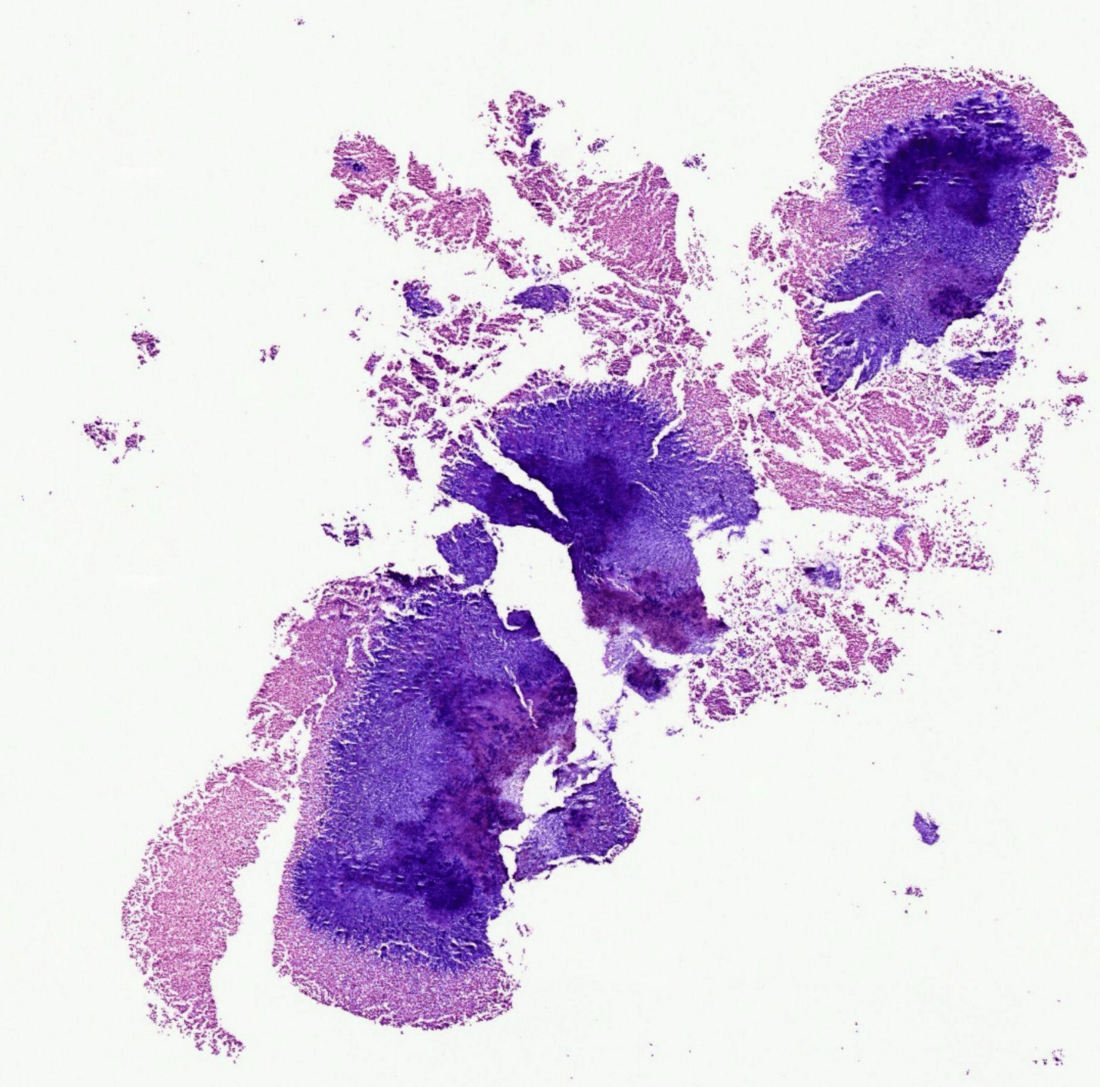
Low-power H&E stain showing basophilic bacterial colonies surrounded by inflammatory infiltrate

**Figure 3:**
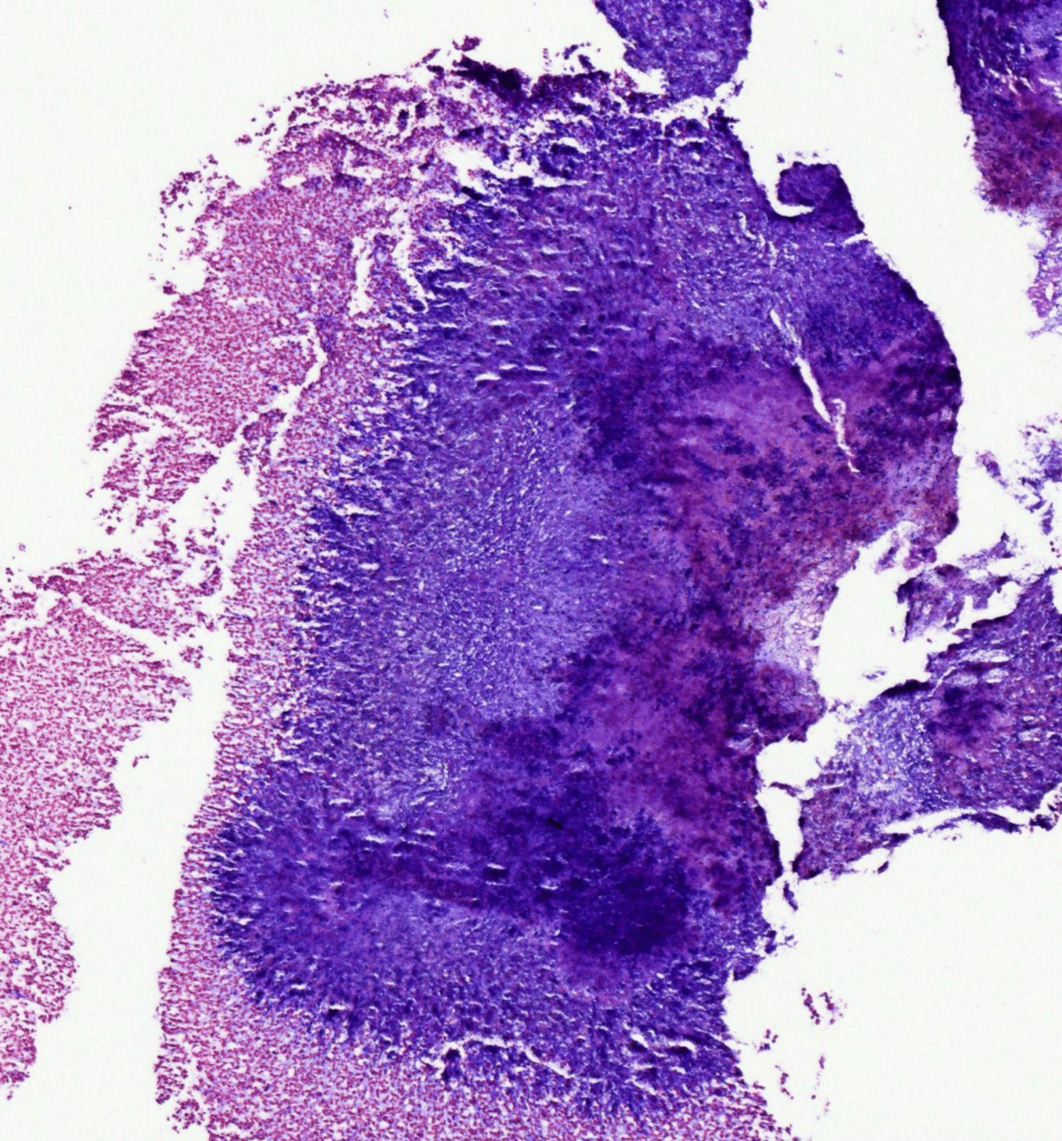
High-power H&E stain highlighting Actinomyces-like filamentous organisms in a central aggregate

**Figure 4:**
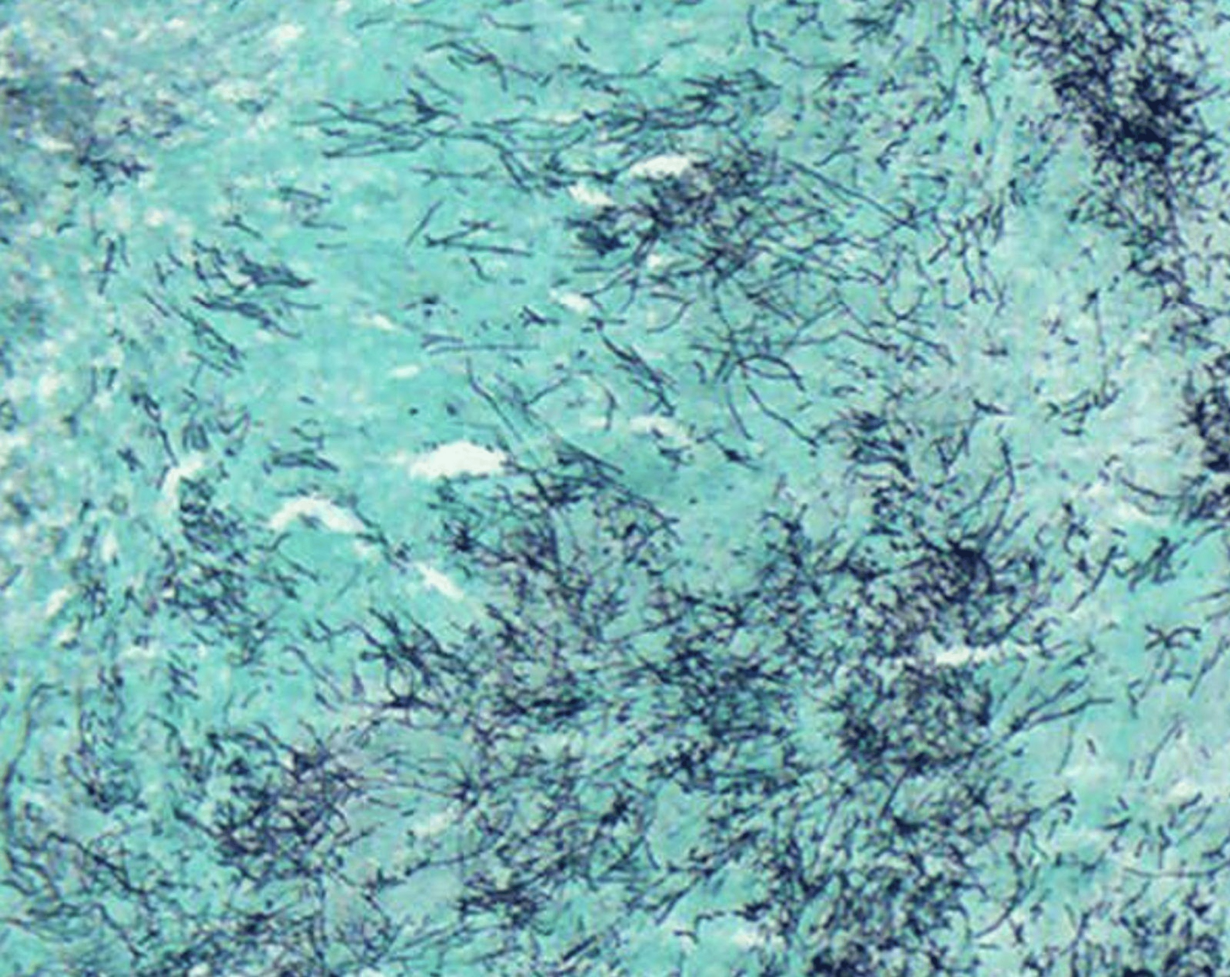
GMS stain confirming filamentous bacterial colonies with characteristic tangled morphology GMS: Grocott Methenamine Silver

Given the prolonged course and unclear diagnosis, a 3 mm punch biopsy was performed. During the procedure, a green-yellow granule was expressed from the dermis, accompanied by a small amount of purulent fluid. Biopsy tissue was submitted for hematoxylin and eosin (H&E) staining (Figures 2-4) and microbial polymerase chain reaction (PCR) testing. Histologic evaluation revealed extensive basophilic filamentous organisms with surrounding granulomatous inflammation, consistent with *Actinomyces* spp. (Figure 2). A special stain demonstrated tangled filamentous colonies of bacteria (Figure 4). Microbial PCR testing detected several commensal skin and oral flora, including *Fusobacterium nucleatum*, *Staphylococcus epidermidis*, *Corynebacterium* spp., and *Cutibacterium acnes*. However, *Actinomyces* was not detected, likely due to its fastidious growth requirements and potential overrepresentation of other flora. These results were interpreted as consistent with contamination or colonization rather than active infection.

At the follow-up one week later, the patient reported new drainage from the lesion but noted reduced tenderness and a slight decrease in lesion size. Examination revealed skin friability and incomplete suture retention. She had not yet started the prescribed amoxicillin-clavulanate due to a pharmacy error, which was corrected during the visit. A new prescription was issued, and she was counseled on the expected course of treatment. Additional recommendations included referral back to oral surgery for dental imaging and further evaluation.

**Figure 5:**
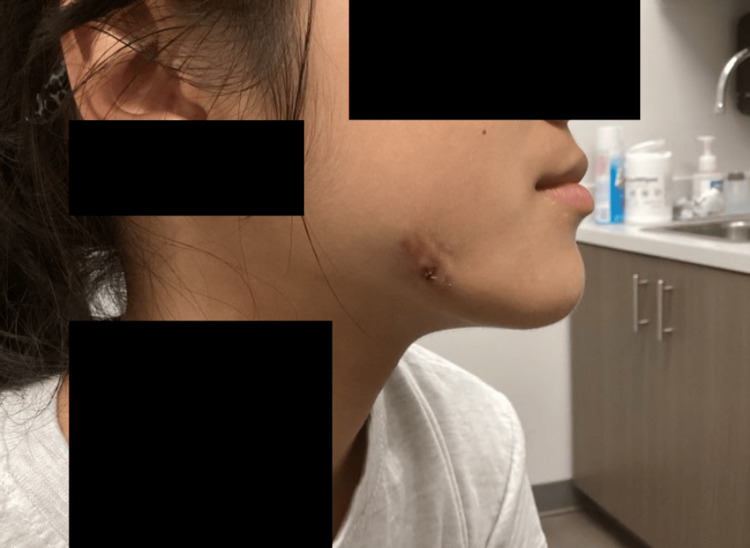
Clinical follow-up photo demonstrating reduction in erythema and induration after initial antibiotic therapy

At the two-week follow-up, the lesion was noted to be clinically improved, with decreased drainage and erythema. The patient completed a three-week course of amoxicillin-clavulanate and was transitioned to long-term amoxicillin 500 mg three times daily for continued management. She had not yet followed up with the oral surgeon at that time. A clinical image obtained at this visit demonstrates continued lesion regression with post-inflammatory erythema (Figure 5).

## Discussion

Cervicofacial actinomycosis remains a diagnostic challenge due to its nonspecific clinical features and its ability to mimic other inflammatory or neoplastic processes [5]. As demonstrated in this case, patients often present with firm, slowly enlarging subcutaneous nodules or abscesses in the setting of recent dental work, which is a common predisposing factor for cervicofacial disease [2,9]. The delay between mucosal disruption and symptom onset, as well as the indolent progression, can complicate timely recognition and treatment [9].

*Actinomyces* species are anaerobic, filamentous, gram-positive bacteria that reside in the oral cavity and become pathogenic when they breach mucosal surfaces [6]. While *Actinomyces israelii* is most frequently implicated, diagnosis rarely relies on species-level identification due to the difficulty of isolating these organisms in culture. The hallmark finding of yellow “sulfur granules,” visible grossly or microscopically, remains one of the most consistent diagnostic clues, especially when microbiologic studies yield only commensal organisms or are entirely negative [5,7].

In our case, histopathologic examination of a punch biopsy revealed filamentous organisms in granule-like clusters with surrounding inflammation, consistent with *Actinomyces* species. This aligns with prior reports noting that tissue biopsy and histopathology often provide a more reliable diagnosis than culture, particularly when samples are exposed to oxygen or prior antibiotic therapy [5,8]. Although our patient’s PCR panel failed to detect *Actinomyces*, this limitation is well-documented due to the organism’s fastidious growth requirements and competition from more abundant skin flora [8]. In this context, histopathologic identification of sulfur granules and filamentous bacteria provided stronger diagnostic weight than PCR results, which may fail to detect *Actinomyces* due to sampling limitations or competition from abundant commensals.

Imaging modalities are often used in cases of cervicofacial actinomycosis to assess for deep extension, including osteomyelitis of the mandible [3]. While our patient had only a superficial lesion, other reports describe imaging features such as soft tissue swelling, sinus tract formation, and bony erosion, particularly when the diagnosis is delayed [2,3].

The initial treatment of cervicofacial actinomycosis traditionally involves high-dose penicillin or amoxicillin for prolonged durations, often up to 6-12 months [6]. However, newer case series suggest that shorter courses may be effective, particularly when the infection is localized and surgical drainage or debridement has been achieved [4]. Our patient responded well to a combination of short-term amoxicillin-clavulanate followed by a prolonged course of amoxicillin, consistent with evolving evidence supporting de-escalation in appropriate clinical contexts [4].

Importantly, this case also emphasizes the value of dermatologic evaluation and biopsy in cases of chronic cutaneous lesions. While cervicofacial actinomycosis is often managed by dental or ENT specialists, the initial diagnosis here was made through skin biopsy in a dermatology clinic, underscoring the critical role dermatologists may play in identifying deep infections masquerading as superficial or neoplastic lesions [1].

## Conclusions

Cervicofacial actinomycosis is a rare but important diagnostic consideration in patients presenting with persistent facial nodules, particularly in the context of recent dental manipulation. The indolent nature of the infection, combined with its ability to mimic neoplastic, granulomatous, or other infectious etiologies, often leads to misdiagnosis or delayed treatment. This case highlights the utility of dermatologic biopsy as a frontline diagnostic tool, especially when more invasive or deep-tissue evaluations are not immediately pursued. Histopathology remains a cornerstone in diagnosing actinomycosis, particularly when microbiologic cultures or molecular studies are inconclusive, as was the case here. The identification of filamentous basophilic bacterial aggregates and sulfur granules on biopsy enabled a definitive diagnosis despite the absence of *Actinomyces* on PCR. 

Timely initiation of antibiotic therapy is essential for the resolution and prevention of complications, such as chronic sinus tract formation or mandibular osteomyelitis. In this case, clinical improvement following targeted antibiotic therapy supports the idea that individualized treatment based on disease extent and clinical response may be appropriate. Ultimately, this case reinforces the need for multidisciplinary awareness; dermatologists, dentists, and infectious disease specialists alike should remain vigilant for cervicofacial actinomycosis in patients with nonhealing facial lesions, particularly when preceded by dental procedures. Prompt diagnosis and therapy can dramatically improve outcomes in this otherwise protracted and deceptively subtle infection.
